# Seromucinous Cystadenoma Presenting as Endometriosis Complications in a 57-Year-Old Female: A Case Report

**DOI:** 10.7759/cureus.26405

**Published:** 2022-06-28

**Authors:** Benjamin Ilyaev, Maria Levada, Matthew Sison, Rebecca Maysonet, Emmanuella Borukh, Vivian Chung, Yakubmiyer Musheyev

**Affiliations:** 1 Medicine, Hofstra University, Hempstead, USA; 2 Obstetrics and Gynecology, New York Institute of Technology College of Osteopathic Medicine (NYITCOM), Old Westbury, USA; 3 Medicine, New York Institute of Technology College of Osteopathic Medicine (NYITCOM), Old Westbury, USA; 4 Medicine, Yeshiva University, New York, USA; 5 Medicine, Saint John’s University, Jamaica, USA

**Keywords:** seromucinous cyst, myomectomy, salpingectomy, oophorectomy, ovarian cystectomy, ovarian cysts, fibroids, endometrioma, endometriosis

## Abstract

Endometriosis should be considered when a female patient reports symptoms of severe pain/tenderness in the pelvic area associated with a frequent need for urination, bloating, vomiting, or nausea. Clinical suspicion is increased if the patient has a history of endometriosis. However, many patients with endometriosis can be asymptomatic, which is why physicians and providers must keep an open mind and have a broad differential. Examinations that aid in the diagnosis of endometriosis include but are not limited to a pelvic examination, an ultrasound, magnetic resonance imaging (MRI), and an exploratory laparoscopy. In this case study, we present a 57-year-old postmenopausal female patient who presented to her obstetrics and gynecology (OBGYN) physician with hot flashes and an abnormal ultrasound revealing an ovarian cyst. Seventeen years prior, at the age of 40, the patient was found to have endometriosis and endometrial polyps and underwent a left oophorectomy. Due to the patient’s history, symptoms, and current scans, it was assumed that the present cyst was a complication of endometriosis. Ultimately, the cyst, right ovarian cyst wall, right fallopian tube, and uterine fibroids were surgically removed and sent to pathology. Upon further review of the patient’s pathology reports, it was found that the cyst removed was a seromucinous cyst with focal borderline features.

## Introduction

Endometriosis is a common and chronic gynecological disease that impacts the pelvic organs of roughly 10% of women of reproductive age, with the ovary being one of the most affected organs [1]. Ovarian endometriomas (OEs), or ovarian endometriosis cysts, are present in 17%-44% of women with endometriosis [1,2]. Moreover, the recurrence rate of such cysts is reported to be up to 50%, creating major concerns for patients with them [3]. The symptoms and signs of OEs often include pain and tenderness in the pelvic area, frequent urination, vomiting, and nausea [4,5]. Some women are asymptomatic, making some OEs challenging to diagnose [4].

The treatment of endometriosis is vast but usually includes ultrasound-guided aspiration and various surgical procedures such as laparoscopy [2]. Many cases are treated empirically by suppressing ovulation and estrogen production. To date, laparoscopic cystectomy seems to be the most effective treatment for endometriosis [2].

Due to the nature in which endometriosis manifests, it may be misdiagnosed initially. Its symptoms are very similar to other ailments, which makes the differential diagnosis list expansive. The various differential diagnoses include functional cyst, ovarian abscess, serous cystadenoma, epithelial carcinoma, germ cell tumor, pelvic inflammatory disease, and ectopic pregnancy [4]. There are also various non-gynecological diagnoses such as appendicitis, diverticulitis, and/or urinary tract infection (UTI) [4].

Ovarian cysts of various extents of malignancy are often connected to an initial endometriosis diagnosis [6]. One such indeterminate malignancy is seromucinous borderline tumors (SMBTs). These tumors have low-grade malignant potential and include aspects of both serous and mucinous tumors [7].

The average age of patients with SMBTs is 33-44, and 30%-50% of such tumors are associated with endometriosis [8]. Diagnosing an SMBT can be difficult because of its similar immunohistochemical expression patterns to OEs [9]. Moreover, studies have attempted to improve the diagnostic distinction between ovarian carcinomas and gastrointestinal metastases [10].

SMBTs have previously been connected to somatic AT-rich interactive domain 1A gene (ARID1A), a tumor suppressor gene mutation, and ARID1A protein loss [8]. This connection is valuable to establish because ARID1A mutations are identified in atypical endometriosis and endometriosis-related carcinomas, meaning such genetic mutations suggest an early sign of malignant transformation [8]. Furthermore, cases of SMBTs have been found to be correlated with genetic mutations in Kirsten rat sarcoma virus (KRAS), while having no substantial connection to phosphatase and tensin homolog (PTEN) mutations [8,11]. Understanding the immunohistochemistry of SMBTs, and endometrioid carcinomas, can allow clinicians to identify patients at a higher risk of carcinogenesis [8]. SMBTs generally have good patient outcomes; thus, correctly diagnosing the tumor, and distinguishing it from OEs and other more aggressive carcinomas, is important for the proper treatment and reduction of aggressive therapies [7].

This article was previously presented as a poster at the 2022 Symposium University Research and Creative Expression (SOURCE) on April 29, 2022.

## Case presentation

A 57-year-old postmenopausal nulligravida female presented to her obstetrics and gynecology (OBGYN) physician with complaints of hot flashes and to counsel for an abnormality found in an ultrasound two months prior that revealed a right adnexal visualized cystic lesion.

She had a family history of stroke, which her mother was diagnosed with at the age of 67. Her social history indicated nothing of significance as to diet, exercise, and stress levels. Her past medical history was significant for endometriosis with endometrial polyps and fibroids and surgical history of a left oophorectomy 17 years prior. Physical examination of the pelvis and genitalia revealed palpable cystocele and rectocele. Laboratory work including CBC, urine analysis, hormone evaluation, Pap smear, and tumor markers (CA-125 and hCG) were all negative and within the normal range. The only abnormality was a slightly elevated HbA1c level.

A pelvic MRI was done a month after the appointment. The test revealed a right ovarian cyst that was slightly larger in comparison to previous ultrasounds conducted. The patient was recommended to have the cyst removed laparoscopically.

The patient was reluctant to undergo another surgery for the treatment of the right ovarian cyst, citing that she had no pain or other symptoms from the cyst. Ultimately, the patient went to see an endometriosis specialist two years later.

The differential diagnosis for a patient with a history of endometriosis and a visualized cyst on an ovary is consistent with an endometrioma.

The patient underwent additional transabdominal and transvaginal ultrasounds of the pelvis one year after the initial presentation. These findings included an anterior myoma at the fundus measuring 3 × 2.5 × 3.2 cm, a left anterior myoma measuring 1.8 × 1.7 × 2.2 cm, and a fundal subserosal myoma measuring 4.3 × 3.7 × 3.6 cm. A right ovarian cyst measuring 6.3 × 4.9 × 6.8 cm and a smaller cyst measuring 1.3 × 0.7 × 1.1 cm were also found. The physician noted that the cyst of the right ovary was larger compared to previous ultrasound findings. The patient also underwent an MRI scan of the pelvis with and without intravenous gadolinium two years after initial presentation. These findings included a complex right ovarian cyst measuring 6.8 × 6 × 6.5 cm and a right fundal exophytic fibroid measuring 5.3 × 4.5 × 4.4 cm, which was found to be stable. Both sets of imaging (MRI and ultrasound) were found to be consistent with an endometrioma.

**Figure 1:**
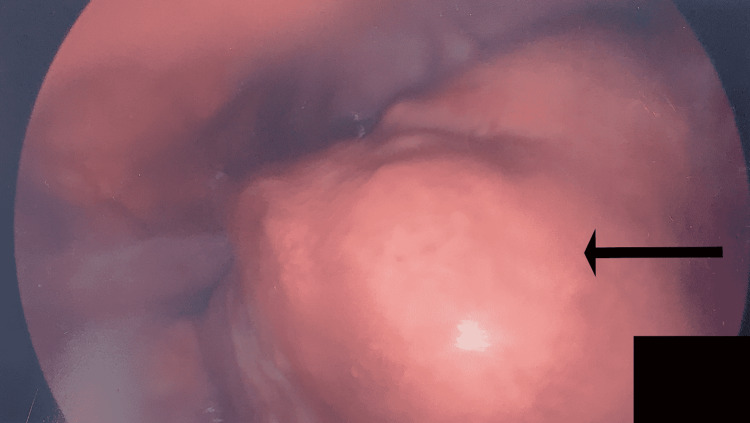
Arrow points toward the 6 × 7 cm fundal fibroid located on the patient’s irregularly enlarged uterus.

**Figure 2:**
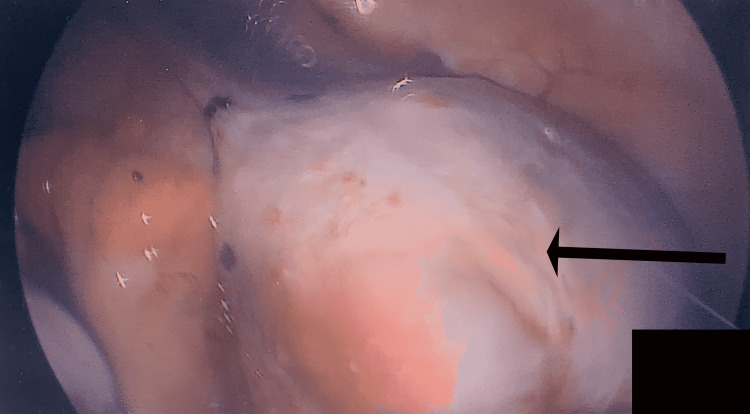
Arrow points toward the 6 × 8 cm cyst located superior to the pelvic brim.

The patient’s preoperative diagnosis was a complex right ovarian cyst. The patient was scheduled for a laparoscopic right cystectomy and oophorectomy, right salpingectomy, removal of adhesions, and myomectomy. The procedure was conducted under general anesthesia. Upon pelvic visualization, the uterus was noted to be irregularly enlarged with one 6 × 7 cm fundal fibroid (Figure 1). Further inspection revealed multiple adhesions of the right ovary, composed of smaller intramural and subserosal fibroids. The location of the ovarian cyst was noted to be superior to the pelvic brim. The cyst was measured to be 6 × 8 cm with a very thick capsule. The excision of the fundal fibroid and the removal of the adhesions were conducted prior to the removal of the ovarian cyst (Figure 2). The right fallopian tube was also excised. Following procedure termination, the patient was brought to the recovery room in stable condition. Specimens sent to pathology included the right ovary, right ovarian cyst, right fallopian tube, uterine fibroid, and adhesions. Intraoperative diagnoses were a right ovarian cyst, fibroid uterus, and pelvic adhesions. Postoperative diagnoses from pathology reported a 5 × 4 × 2 cm seromucinous cystadenoma, with focal borderline features. Uterine fibroid samples were found to be benign leiomyoma, and the samples from the fallopian tube were deemed unremarkable.

The preferred therapy for a cyst (suspected to be a complication of endometriosis) is surgical removal [12]. In this case, the surgeon performed a laparoscopy and was able to conduct a right salpingectomy, right ovarian cystectomy, and right oophorectomy. Following the operation, the patient was kept overnight at the hospital in order to continuously monitor her vitals. The patient was discharged the following day, and a follow-up appointment was set for one week after the surgery. At the follow-up, the patient was noted to be doing well and healing/recovering adequately.

## Discussion

Symptoms of endometriosis may present in various ways that mimic other conditions, making its diagnosis and treatment pertinent to patient health. Its symptoms include pain or pressure in the lower abdomen and severe acute pain along with nausea and vomiting if the cyst has ruptured [4]. In order to diagnose endometriosis, the clinician may use health history, physical examinations, blood work, and imaging such as MRI or CT scans, as used in the case presented [4]. Recurrence of endometriomas is another problem that arises with the treatment of endometriosis. To properly manage the disease, a prediction of the patient’s risk must be determined, as this will ensure proper treatment is being implemented for the patient [3]. One of the more frequent diagnoses associated with endometriosis is the borderline seromucinous cyst. Due to its similarity in presentation, many cases of borderline seromucinous cysts go undiagnosed or misdiagnosed, furthering its course [9].

Borderline seromucinous cysts present in manners similar to endometriosis, sharing similar symptoms as well. In the case presented, the patient was believed to have a recurrent case of endometriomas. This was suggested by presentation, past medical history, and medical imaging. Ultimately, upon surgical intervention and pathological examination, the diagnosis was found to be borderline seromucinous cysts. It is interesting to note that there have been other documented cases highlighting how seromucinous borderline tumors were confused for an endometrioma. During a similar case report, a nontender mass was discovered while a physical examination of a 39-year-old pregnant Japanese patient was conducted [9]. After an initial sonogram, the mass was thought to be ovarian cancer; however, after further imaging through an MRI examination, the mass was thought to be a decidualized endometrioma [9]. Unlike the patient presented in this case study, the 39-year-old patient had not had any past medical history of endometriosis, pain in the abdominal region, or abdominal surgery [9]. Only after surgical intervention and receiving the histopathological results was the cyst discovered to be a seromucinous borderline tumor [9].

As seromucinous cysts are frequently borderline, the risk of serious outcomes is small [8]. Because of the patient’s past medical history and negative tumor marker tests, there was an initial assumptive diagnosis of a benign endometrial cyst. As a result, against the doctor’s recommendation, the patient did not feel immediate removal necessary, allowing for the patient to prolong surgical intervention. After the surgical intervention and subsequent pathology reports, it was discovered that the diagnosis was in fact a seromucinous cystadenoma with focal borderline features. It became clear that surgical removal was quite indicated. It is valuable to consider all differentials when diagnosing in order to inform the patient of all possibilities that could arise and what could occur if one delays treatment.

## Conclusions

The symptoms, modes of diagnosis, and various treatments involved with endometriosis were discussed in this case study. The signs and symptoms that are used to diagnose seromucinous cysts were also examined.

In this case study, a female patient presented with what appeared to be signs of endometriosis but ultimately ended up being a seromucinous cyst with borderline features. As previously stated, in this case, because it was assumed that the right ovarian cyst was a recurrent endometrioma, the surgery was delayed by the patient, allowing the seromucinous cyst a chance to grow. This is clinically relevant because it shows just how important it is to keep in mind a broader differential diagnosis in order to avoid possible negative outcomes and for physicians to provide the best possible treatment for their patients.
